# Predicting deep vein thrombosis using machine learning and blood routine analysis

**DOI:** 10.3389/fdata.2025.1605258

**Published:** 2025-10-06

**Authors:** Jie Su, Yuechao Tang, Yanan Wang, Chao Chen, Biao Song

**Affiliations:** ^1^Medical Neurobiology Laboratory, Inner Mongolia Medical University, Hohhot, China; ^2^Inner Mongolia Health Digital Society, Hohhot, China; ^3^Baoding Second Central Hospital, Baoding, China; ^4^Medical Intelligent Diagnostics Big Data Research Institute, Hohhot, China; ^5^Inner Mongolia University of Finance and Economics, Hohhot, China

**Keywords:** deep vein thrombosis, machine learning, blood routine, prediction model, SHAP analysis

## Abstract

**Objective:**

Lower limb deep vein thrombosis (DVT) is a serious health problem, causing local discomfort and hindering walking. It can lead to severe complications, including pulmonary embolism, chronic post-thrombotic syndrome, and limb amputation, posing risks of death or severe disability. This study aims to develop a diagnostic model for DVT using routine blood analysis and evaluate its effectiveness in early diagnosis.

**Methods:**

This study retrospectively analyzed patient medical records from January 2022 to June 2023, including 658 DVT patients (case group) and 1,418 healthy subjects (control group). SHAP (SHapley Additive exPlanations) analysis was employed for feature selection to identify key blood indices significantly impacting DVT risk prediction. Based on the selected features, six machine learning models were constructed: k-Nearest Neighbors (kNN), Logistic Regression (LR), Decision Tree (DT), Random Forest (RF), Support Vector Machine (SVM), and Artificial Neural Network (ANN). Model performance was assessed using the area under the curve (AUC).

**Results:**

SHAP analysis identified ten key blood routine indices. The six models constructed using these indices demonstrated strong predictive performance, with AUC values exceeding 0.8, accuracy above 70%, and sensitivity and specificity over 70%. Notably, the RF model exhibited superior performance in assessing the risk of DVT.

**Conclusions:**

Our study successfully developed machine learning models for predicting DVT risk using routine blood tests. These models achieved high predictive performance, suggesting their potential for early DVT diagnosis without additional medical burden on patients. Future research will focus on further validation and refinement of these models to enhance their clinical applicability.

## 1 Introduction

DVT is a form of venous thromboembolic (VTE) disease, characterized by abnormal coagulation of blood within the deep veins, resulting in either complete or partial vascular obstruction. DVT predominantly affects the deep veins of the lower and upper extremities but can also manifest in the deep veins of the upper limbs, visceral veins, and vena cava (Lyman et al., 2021). The prevalence of venous thrombosis is surpassed only by that of coronary heart disease and hypertension among cardiovascular diseases, with its mortality rate ranking second to that of tumors and myocardial infarction. The United States records over 300,000 DVT cases annually, with the risk escalating to 1 in 100 for individuals aged > 50 years (Heit, 2006).

Current DVT diagnosis relies on a combination of clinical symptoms, imaging, and laboratory (Knudson et al., 1992; Righini et al., 2008; Shaikhouni et al., 2018). However, atypical presentations, symptom variability, and high rates of asymptomatic cases often lead to delayed or missed diagnoses, creating significant clinical challenges (Knudson et al., 1992; Shaikhouni et al., 2018). Early risk prediction and targeted prevention are therefore critical to reducing morbidity and mortality associated with DVT.

Machine learning (ML) has revolutionized medical diagnostics and risk stratification across diverse conditions, including cardiovascular diseases (CVDs), cancer, and chronic illnesses (Deng et al., 2023; Xu and Xu, 2024; Zhang et al., 2025; Zhong et al., 2019). In CVDs, hybrid models integrating recurrent neural networks (RNNs) and long short-term memory (LSTMs) have improved ECG-based detection, while deep learning approaches incorporating genetic and lifestyle data enhance risk prediction (Krones et al., 2025; Maurovich-Horvat, 2021). Similarly, ML algorithms like convolutional neural networks (CNNs) have advanced cancer diagnostics by analyzing medical images (Rabiei et al., 2022), and RF models have enabled accurate brain tumor prediction by integrating clinical and genetic data (Alzubi et al., 2019). For chronic diseases such as chronic kidney disease (CKD) and diabetes, ML facilitates early risk identification and personalized management (Schena et al., 2022), while optimized feature selection techniques have enhanced COVID-19 diagnostic accuracy (Braik et al., 2023).

Despite significant advancements in DVT prediction research, several critical limitations remain: current models rely heavily on imaging examinations or invasive biomarker testing, limiting their applicability in primary care settings and resource-limited regions; there is a lack of systematic performance comparison studies of prediction models across different clinical scenarios; furthermore, the underutilization of low-cost, highly accessible routine tests such as complete blood count severely restricts clinical translation and large-scale application of these predictive models. This study addresses these gaps by focusing on DVT prediction using routine blood data, aiming to develop an accessible, cost-effective tool for early risk stratification.

## 2 Related work

Recent years have witnessed growing adoption of ML for DVT prediction, with studies focusing on diverse patient populations and algorithms. Table 1 summarizes key findings, highlighting variations in cohorts, algorithms, and performance.

**Table 1:** Comparative analysis of prediction models for DVT.

**Research team**	**Model characteristics**	**DVT-specific innovations**	**Limitations**
(Jin et al. 2022)	Compared 5 algorithms (LR+D-dimer optimal)	First cancer-associated DVT nomogram & web calculator	Limited to oncology patients
(Ding et al. 2023)	XGBoost (AUC 0.89)	Identified bilirubin as novel DVT biomarker	Single-center arthroplasty data
(Deng et al. 2023)	BRIDGE model (Logistic Regression)	Post-transplant DVT mortality prediction system	Mortality-focused (not DVT incidence)
(Ryan et al. 2021)	Gradient Boosting (12–24 h pre-onset)	Ultra-early DVT warning framework	Short-term prediction only
(Liu et al. 2021)	SVM (Sensitivity 92%)	Age-specific DVT prediction for young inpatients	Excludes elderly populations
Liu L. et al. (2024)	Random Forest (Specificity 88%)	Dynamic LE-DVT risk assessment post-stroke	Requires periodic imaging confirmation
(Wu et al. 2024)	Random Forest (Internal AUC 0.91)	Spinal surgery DVT complication alert system	Lacks multicenter validation
(Wei et al. 2024)	XGBoost (F1-score 0.85)	Integrated DVT prediction-prevention for fractures	External validation pending
Liu X. et al. (2024)	XGBoost (NRI 0.21)	CRC perioperative DVT risk stratification tool	Not generalized to other cancers
(Nothnagel and Aslam 2024)	AI-POCUS system (κ=0.79)	First telemedicine DVT diagnostic AI platform	Requires specialized ultrasound equipment

Despite the promise of machine learning models in DVT prediction, significant limitations remain: most studies focus on narrow populations (e.g., post-surgical or cancer patients), limiting generalizability; reliance on specialized data (e.g., surgical metrics, cancer history) restricts application in primary care or low-resource settings; and many models lack external validation, hindering clinical translation. Additionally, low-cost, widely accessible routine data (e.g., blood routine tests)—despite their potential for broad implementation—remain underutilized. This study addresses these gaps by developing a DVT prediction model based on routine blood data, aiming to provide an accessible tool for early risk assessment across diverse clinical scenarios.

## 3 Methods and materials

### 3.1 Data collection

For this research, a dataset was gathered from patients hospitalized at the Baoding Second Central Hospital from January 2022 to June 2023. Ethical approval for this retrospective study was obtained from the Ethics Committee of Baoding Second Central Hospital, and the requirement for informed consent was waived. The study was conducted in accordance with the Declaration of Helsinki.

The inclusion criteria were as follows:

1. Age between 50 and 80 years old.
2. Hospitalized in general or thoracic surgery department.
3. No other serious cardiovascular diseases or bleeding tendencies.

The exclusion criteria were as follows:

1. Incomplete routine blood data.
2. Test data exceeding the reference range by more than 10-fold.
3. Other factors affecting hemorheology (e.g., recent surgery, ongoing anticoagulant therapy).

**Figure 1:**
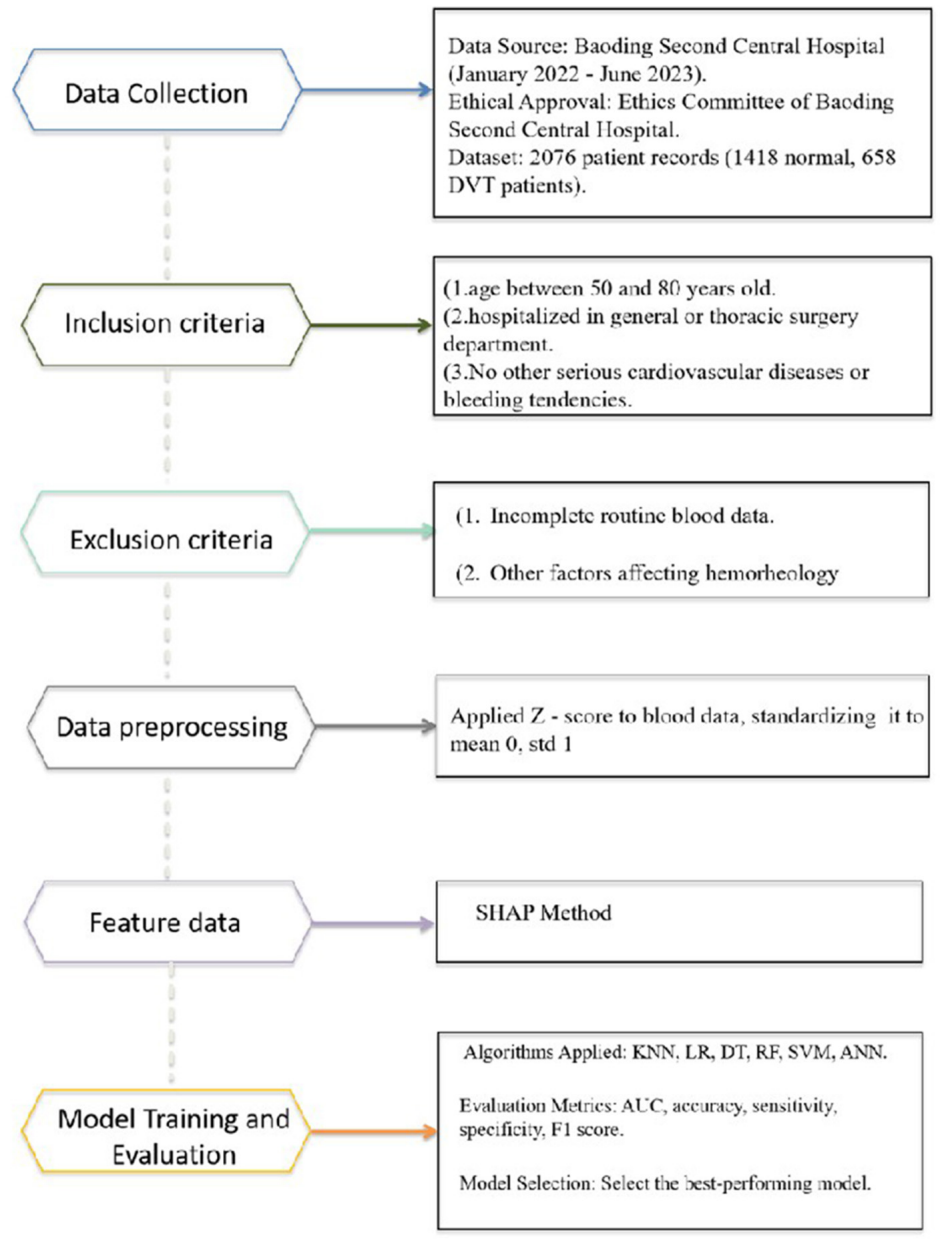
Flowchart of the ML development model for predicting DVT.

We extracted the basic information and routine blood data of patients from the hospital information system, and constructed a dataset with patients with DVT as positive samples and patients without DVT as negative samples, containing 28 attributes. The detailed information on all attributes is provided in Tables 2, 3. The dataset contained 2,076 patient records, of which 1,418 (68.3%) were normal and 658 (31.7%) had DVT. The data screening process is illustrated in Figure 1.

**Table 2:** Patient demographics descriptions for DVT study.

**English name**	**Abbreviation**	**Description**	**Unit/Range**
Age	Age	Age of the patient	Years
Gender	Gender	Gender of the patient (1 for male, 0 for female)	Categorical (1/0)
Department	Dept	Hospital department (varied DVT incidence rates across departments)	Categorical
Category	Cat	Patient type (inpatients/outpatients; inpatients at higher DVT risk due to limited mobility/long bed rest)	Categorical (inpatient/outpatient)
Clinical diagnosis	CD	Clinical diagnosis of the patient	DVT
Blood routine test time	BRTT	Time of the patient's routine blood test	Date/time

**Table 3:** Patient blood test parameter descriptions for DVT study.

**English name**	**Abbreviation**	**Description**	**Unit/range**
Red blood cell count	RBC	Number of red blood cells in the blood	10^12^/L; normal range: 4.3–5.8 (male), 3.8–5.1 (female)
Mean corpuscular volume	MCV	Average volume of a single red blood cell	fL, normal range is 82–100
Platelet distribution width	PDW	Variation in the size distribution of platelets in the blood	fL, normal range is 9–17
White blood cell count	WBC	Number of white blood cells in the blood	10^9^/L; normal range: 3.5–9.5
Neutrophil percentage	NEUT%	Proportion of neutrophils in white blood cells	%; normal range: 40–75
Lymphocyte percentage	LYMPH%	Proportion of lymphocytes in white blood cells	%; normal range: 20–50
Eosinophil percentage	EO%	Proportion of eosinophils in white blood cells	%, normal range is 0.4–8
Basophil percentage	BASO%	Proportion of basophils in white blood cells	%, normal range is 0–1
Neutrophil absolute value	NEUT	Absolute number of neutrophils in the blood	10^9^/L; normal range: 1.8–6.3
Lymphocyte absolute value	LYMPH	Absolute number of lymphocytes in the blood	10^9^/L; normal range: 1.1–3.2
Basophil absolute value	BASO	Absolute number of basophils in the blood	10^9^/L; normal range: 0–0.06
Hemoglobin	HGB	Content of hemoglobin in the blood	g/L; normal range: 130–175 (male), 115–150 (female)
Hematocrit	HCT	Proportion of red blood cells in the total volume of blood	Unitless; normal range: 0.4–0.5 (male), 0.35–0.45 (female)
Mean corpuscular hemoglobin	MCH	Content of hemoglobin in a single red blood cell	pg; normal range: 27–34
Mean corpuscular hemoglobin concentration	MCHC	Content of hemoglobin per unit of blood	g/L; normal range: 316–354
Red cell distribution width	R-CV	Variation in the size distribution of red blood cells in the blood	%; normal range: 10.9–15.4
Platelet count	PLT	Number of platelets in the blood	10^9^/L; normal range: 125–350
Mean platelet volume	MPV	Average volume of a single platelet	fL; normal range: 9–13
Plateletcrit	PCT	Proportion of platelets in the total volume of blood	%; normal range: 0.11–0.28
Monocyte absolute value	MONO	Absolute number of monocytes in the blood	10^9^/L; normal range: 0.1–0.6
Monocyte percentage	MONO%	Proportion of monocytes in white blood cell	%; normal range: 3–10
Eosinophil absolute value	EO	Absolute number of eosinophils in the blood	10^9^/L; normal range: 0.02–0.52

### 3.2 Data preprocessing

To eliminate the differences in the dimensions and magnitudes between different routine blood indicators, we used the Z-score normalization method to preprocess the data. The Z-score normalization method (Zhang and Chen, 2012) transforms the original data into standard normal distribution data with a mean of 0 and a standard deviation of 1. The transformation formula is as follows:


$$\begin{array}{l} \text{z}=\frac{\text{x }-\mu }{\sigma } \end{array}$$

Here, Z represents the standardized data, X is the original data, μ is the mean of the original data, and σ is the standard deviation of the original data. Through Z-score normalization, data can be made comparable, while reducing the impact of outliers and noise. This process enhances the robustness of the machine learning model against variations in the data.

To address the issue of class imbalance in the dataset, this study employed the Synthetic Minority Oversampling Technique (SMOTE) for oversampling, aiming to optimize the class distribution of training data and mitigate potential biases of the model toward the majority class. This approach is more methodologically sound compared to random oversampling (which tends to induce overfitting) and undersampling (which risks information loss). By generating synthetic samples through interpolation between minority-class instances, SMOTE not only effectively balances class distribution but also preserves the feature structure of the original data.

To validate the performance of the proposed algorithm, an independent test set was constructed in this study by extracting 69 samples from both the experimental group and the control group, resulting in a 1:1 ratio between the two groups within the independent test set. 5-fold cross-validation was introduced: the remaining samples was randomly divided into five mutually exclusive subsets, which were used for iterative training and validation. The mean performance across the different validation subsets was adopted as the final model performance metric.

### 3.3 Feature selection

#### 3.3.1 SHAP method

For feature selection, we used the SHAP method proposed by (Lundberg et al. 2020), the aim of which was to filter out the most beneficial related features from the original data. This increased the efficiency and accuracy of the model, reduced running time, and enhanced the interpretability of the model. The core aim of SHAP is to calculate the SHAP values of each feature, which reflect the contribution of the feature to the predictive ability of the entire model. Specifically, SHAP interprets the prediction value of the model as the sum of the attribution values of each input feature, that is,


$$\begin{array}{l} f\left(x\right)=\emptyset_{0}+\overset{N}{\underset{i=1}{\sum }}\phi_{i}•x_{i} \end{array}$$

Here, ∅_0_ is the base value, N is the total number of features, *x*_*i*_ is the actual value of the ith feature, and ∅_*i*_ represents the SHAP value of the ith feature, reflecting the impact of this feature on the output of the model. The SHAP calculation involves taking a weighted average of the predicted values of all possible subsets S of features to obtain the contribution of each feature under different feature combinations. The definition formula for the Shapley value is


$$\begin{array}{l} \phi_{i}\left(f\right)=\sum_{S\subseteq N\backslash \left\{i\right\}}\frac{|S|!\left(|N|-|S|-1\right)!}{|N|!}\left[f\left(S\cup \left\{i\right\}-f\left(S\right)\right)\right] \end{array}$$

This formula indicates that the Shapley value of the i-th feature is a weighted average over all subsets S of the features, where the weights are determined by the size of the subset and the order of the features. *f* (S∪) (Olaf and Cooney, 2017) represents the prediction of the model when both the features in S and the i-th feature are present, whereas *f* (S) is the prediction of the model when only the features in S are present.

#### 3.3.2 Feature ranking process

##### 3.3.2.1 SHAP value calculation

We used the SHAP library to compute the SHAP values for all commonly used clinical test items included in our study. The SHAP value, which is based on the Shapley value concept in game theory, can precisely quantify the contribution of each feature to the model's prediction results. For each sample, the SHAP value represents the average marginal contribution of a particular feature to the prediction result across all possible feature combinations. Through calculations of a large number of samples, we obtained the distribution of the SHAP values corresponding to each feature.

##### 3.3.2.2 Sorting by mean absolute value

To rank the features, we used the mean of the absolute values of SHAP for all samples as the criterion for determining the feature importance. The reason for using the mean of absolute values is that the larger the absolute value of the SHAP value, the more significant is the impact of that feature on the model's prediction results, regardless of whether the impact is positive or negative. After arranging all features in descending order according to the mean of the SHAP absolute values, we successfully obtained the importance ranking of the features. For example, in the dataset of our study, the mean of the absolute value of SHAP for red blood cells (RBC) ranked among the highest of all features. This clearly shows that RBC are an extremely crucial feature of DVT diagnostic models.

### 3.4 Performance evaluation metrics

Six classification algorithms were applied to the preprocessed dataset to determine the best performer algorithms by comparing the AUC, accuracy, and other statistical measures. The algorithms applied were the KNN, LR, DT, RF, SVM, and ANN. A brief overview of the performance evaluation is provided in this subsection.

(1) The sensitivity, specificity, and accuracy of each algorithm's results were calculated using a confusion matrix.

$$\begin{array}{l} sensitivity\;=\frac{TP}{TP+FN}\\ sensitivity\;=\frac{TN}{TN+FP}\\ accuracy\;=\frac{TP+TN}{TP+FP+TN+FN} \end{array}$$

Here, TP represents true positives, which are the samples predicted as positive and actually positive; TN represents true negatives, which are the samples predicted as negative and actually negative; FP represents false positives, which are the samples predicted as positive but actually negative; FN represents false negatives, which are the samples predicted as negative but actually positive.

(2) The receiver operating characteristic (ROC) curve is a graph with the false-positive rate (FPR) on the x-axis and the true positive-rate (TPR) on the y-axis. The ROC curve represents the predictive performance of a model at different thresholds, and is suitable for comparing the merits of different models. The calculation formulas for the FPR and TPR are as follows:

$$\begin{array}{l} FPR=\frac{FP}{FP+TN}\\ TPR=\;\frac{TP}{TP+FN}\; \end{array}$$

(3) AUC refers to the area under the ROC curve. This reflects the degree of closeness between the model's predicted probability distribution of positive samples and the actual probability distribution of positive samples. The larger the value, the better is the performance of the model. AUC is suitable for assessing the overall performance of a model.
(4) The F1-Measure is a critical metric for evaluating classification model performance, especially in class-imbalanced scenarios. It is derived from the harmonic mean of precision and recall, quantifying their trade-off. Ranging from 0 to 1, a higher F1-Measure indicates a better balance between the two. Its mathematical formula is:

$$\begin{array}{l} \text{F1-Measure}=2*\frac{\text{Precision}\times \text{sensitivit}}{\text{Precision}+\text{sensitivit}} \end{array}$$

### 3.5 Supervised ML algorithms

In this study, we employed distinct hyperparameter optimization strategies tailored to the specific needs of different machine learning algorithms. For non-artificial neural network (non-ANN) algorithms, we utilized Grid Search. This method systematically explores a predefined set of hyperparameters by evaluating all possible combinations to identify the configuration that maximizes model performance. Key parameters of each algorithm were meticulously defined as search dimensions to ensure comprehensive optimization. For ANNs, given their complexity and the vast parameter space, we adopted a Random Search approach. We pre-specified a range for the number of hidden layers and neurons per layer, and conducted at least 10 random search iterations to determine the optimal architectural parameters. The model was trained with a fixed epoch number of 100, and an early stopping strategy was employed to prevent overfitting—with parameters set as monitor = val_auc, patience = 20, and mode=max. This approach balances computational efficiency with the need to explore a wide range of potential configurations. This section provides a concise overview of the fundamental principles of supervised machine learning and details the optimal hyperparameters identified through these strategies for each model.

(1) The KNN (Siddalingappa and Kanagaraj, 2022; Vaisali et al., 2025) algorithm calculates the distance between the sample to be classified and labeled training samples. It identifies the K training samples that are nearest in distance as references, and assigns a label to the sample to be classified based on these labels. A distinctive characteristic of the KNN algorithm is its ability to make predictions directly using the training data, without the need for explicit model learning. This approach is often referred to as instance-based or lazy learning. For this specific dataset analysis, the optimal parameters for the dataset were determined to be an n_neighbors value of 7, a brute algorithm, and a *p*-value of 1, with all other parameters set to their default values.

(2) The LR (Menard, 2013; Uddin et al., 2019) transforms the outcomes of linear regression into a probability ranging between 0 and 1 via an S-shaped log-odds function, subsequently determining the sample category based on this probability. The LR parameters can be derived by maximizing the log-likelihood function, with gradient descent and Newton's method being the most prevalent optimization algorithms used for this task. For the analysis of this dataset, the optimal parameters were identified as solver=saga and random_state=168, with all other parameters adhering to their default values.

(3) The DT (Cruz and Wishart, 2007; Podgorelec et al., 2002) uses a tree-like decision model to represent the rules for data partitioning and prediction. Each internal node of the decision tree signifies a test for an attribute, each branch denotes the outcome of a test, and each leaf node corresponds to a category. The construction of a decision tree is a top-down recursive process. Starting from the root node, the optimal attribute is chosen to partition the dataset based on a specific evaluation criterion, and this process is repeated for each subset until the termination condition is satisfied. Common evaluation criteria include information gain, gain ratio, and Gini index, which can be used to gauge the purity or uncertainty of an attribute. The attribute that maximizes the purity enhancement or minimizes the uncertainty is selected as the optimal attribute. For the dataset under consideration, the best-fit parameters were determined as criterion=entropy, max_features=auto, max_depth=5, and random_state=126, with all other parameters maintained at their default values.

(4) The RF (Becker et al., 2023; Buri and Hothorn, 2020) enhances classification accuracy and stability by generating multiple decision trees and amalgamating their predictive outcomes. The principal attribute of RF is the incorporation of two forms of randomness: first, during the training of each decision tree, a subset is extracted from the original dataset with replacement via the bootstrap method to serve as the training data; second, when determining each split node, a fraction of the features is randomly chosen from all features to act as candidate features. These dual forms of randomness empower RF to diminish the variance and bias inherent in a single decision tree, bolster the model's generalization capability, and counteract overfitting. For this dataset evaluation, the optimal parameters for the dataset were identified as n_estimators=13, max_depth=5, and random_state=126, with all other parameters retained as their default values.

(5) The SVM (Huang et al., 2018; Wang et al., 2023) operates by identifying a maximum-margin linear classifier within the feature space, segregating samples of varying categories, and maximizing the distance from the nearest sample point (i.e., the support vector) to the classifier. The fundamental concept of the SVM is to maximize the margin, which can be formalized as a convex quadratic programming problem and is tantamount to the minimization problem of the regularized hinge loss function. The SVM can additionally incorporate a kernel function to map the original feature space to a high-dimensional feature space, thereby facilitating nonlinear classification. For the analysis of this specific dataset, the optimal parameters for the dataset were determined as C = 0.9, gamma = 0.1, coef0 = 0.5, random_state=126, and probability=true, with all other parameters retained as their default values.

(6) The ANN (Mousannif et al., 2021; Yamamura, 2003) is a computational model that emulates the neural system of the human brain. ANNs are comprised of multiple neurons, each of which receives numerous input signals. These signals undergo processing via a weighted sum and activation function to generate an output signal, which then serves as the input signal for the subsequent layer of neurons. The ANN training process involves adjusting the network parameters using data with the aim of the network output closely approximating the real labels or target values of the data. Commonly employed ANN methods include gradient descent, stochastic gradient descent, batch gradient descent, the momentum method, and the adaptive learning rate method. For this particular neural network configuration exploration, the network consisted of two hidden layers with 384 and 64 neurons each. The first hidden layer employed l1 regularization with a regularization weight of 0.01. The activation function was ReLU, and the optimization function was Adam, which had a learning rate of 0.0081. The loss function was binary cross-entropy, and the batch size was 107.

In this study, Python software was employed for data processing and model implementation, with the utilization of relevant libraries including TensorFlow, Pandas, NumPy, Imblearn, and Scikit-Learn (sklearn).

## 4 Results

### 4.1 Results of data analysis

**Figure 2:**
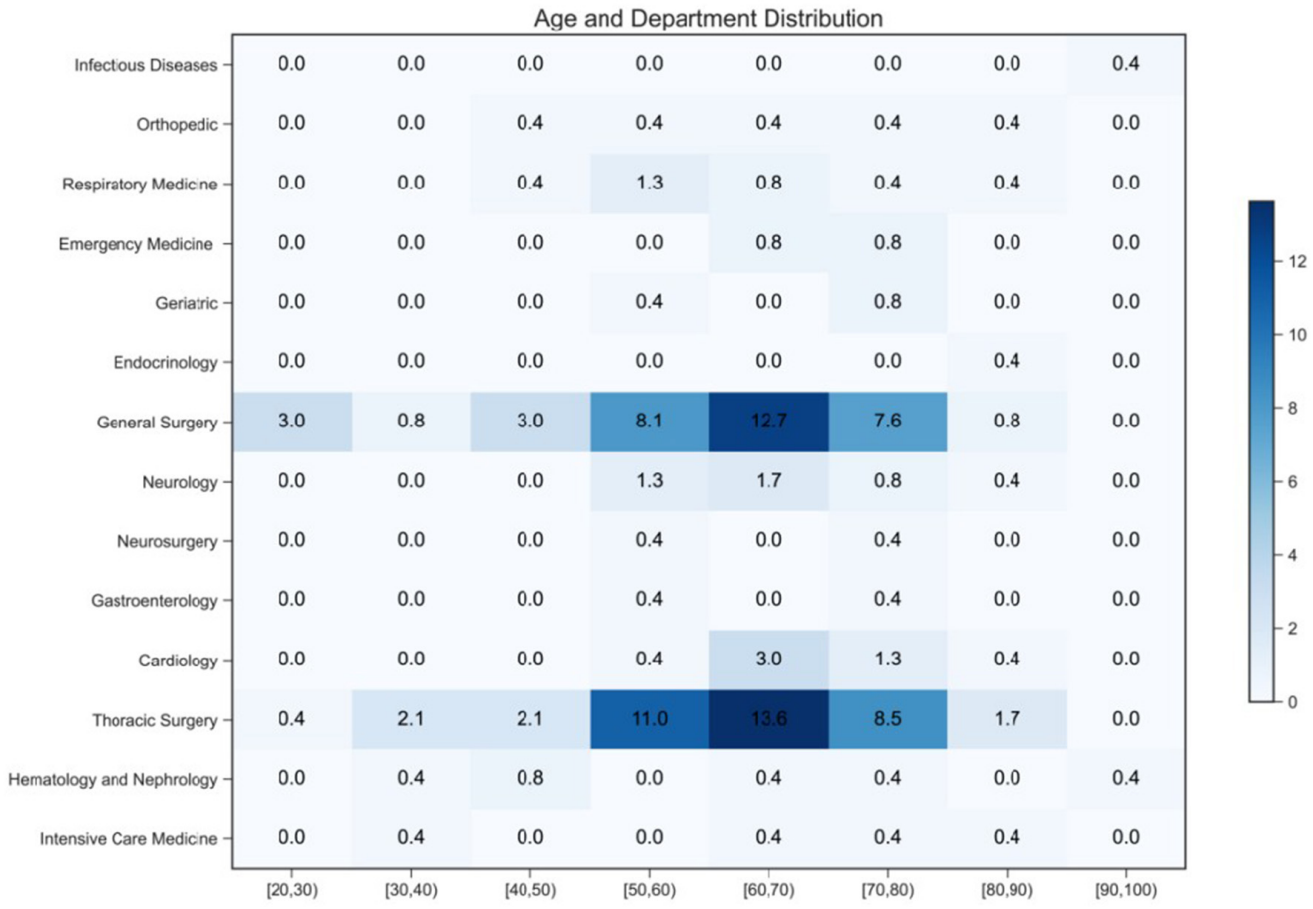
Analysis of the distribution of hospitalized patients with DVT.

To explore the characteristics of patients with lower-extremity venous thrombosis and establish reasonable inclusion criteria, age and department of patients with lower-extremity venous thrombosis who visited the Affiliated Hospital of Inner Mongolia Medical University from January 2022 to June 2023 were statistically analyzed. A heatmap (Figure 2) was drawn, which showed the proportion of patients in different age ranges and who attended different departments. The values in the heatmap represent the percentage of the number of patients in that area to the total number of patients, and the color depth represents the size of the proportion. The results showed that patients with lower-extremity venous thrombosis were mainly concentrated in the age group of 50–80 years old, and the incidence rates in thoracic and general surgery were substantially higher than those in other departments. Therefore, the inclusion criteria were determined as age between 50 and 80 years old and attending as an inpatient in the thoracic surgery or general surgery department.

### 4.2 Results of feature selection

For predicting the risk of lower-extremity venous thrombosis, we used the absolute range of SHAP values to select features, setting a threshold at 0.2. This threshold was chosen to balance “core information coverage” and “model simplicity,” ensuring that the retained features accounted for over 85% of the total contribution while minimizing the risk of overfitting or underfitting.

**Figure 3:**
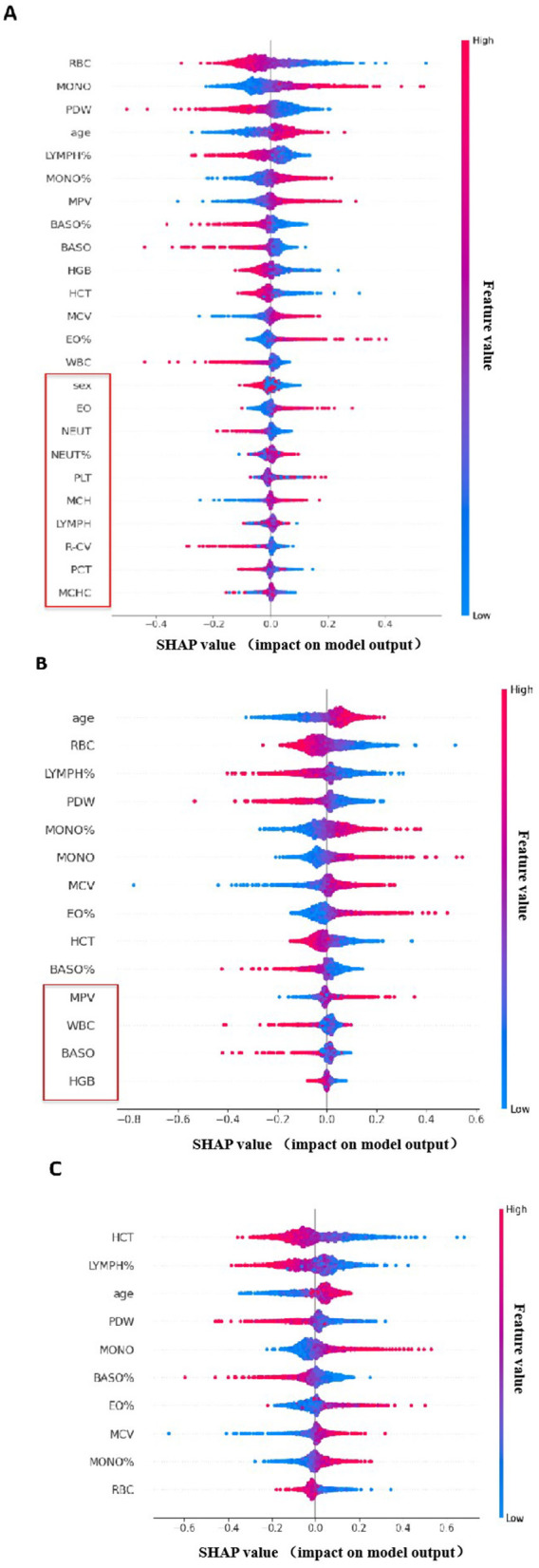
Diagram of the Screening and Optimization Process for SHAP Feature Impact Distribution. **(A)** SHAP Summary Plot of the First-Round Feature Selection. Color Coding: The color gradient from blue to red represents feature values, where blue indicates a low feature value and red indicates a high feature value. X-axis: Denotes the SHAP value, which reflects the impact of each feature on the output of DVT risk prediction model. Features labeled in the plot were excluded in the first round due to their SHAP value range being less than 0.2. **(B)** SHAP Summary Plot of the Second-Round Feature Selection. Low-contribution features highlighted in the plot were removed in the second round to refine the feature set. **(C)** SHAP Summary Plot of the Final-Round Feature Selection. Clinical Annotations for Each Feature: age: Increased age is associated with higher thrombotic susceptibility; RBC: RBC levels are linked to blood viscosity, a known risk factor for DVT; MCV: As an erythrocyte parameter, it is associated with oxygen-carrying capacity, which in turn influences thrombotic risk; PDW: Reflects platelet heterogeneity; abnormal PDW may alter thrombogenic potential; LYMPH%: Associated with inflammatory responses, which play a key role in the pathogenesis of DVT; EO%: Although the association is weak, eosinophils can regulate inflammation processes related to thrombosis; BASO%: Participates in inflammatory and hemostatic pathways associated with DVT; HCT: As an erythrocyte parameter, it is linked to blood viscosity and oxygen-carrying capacity—both of which are related to thrombotic risk; MONO: Monocytes are involved in inflammatory responses and may affect thrombotic processes; MONO%: Similar to MONO, monocyte percentage reflects the inflammatory status relevant to DVT.

We conducted two rounds of feature selection (Figure 3). The first round excluded 10 features (NEUT%, NEUT, LYMPH, MCH, MCHC, R-CV, PLT, PCT, Sex, EO) from an initial set of 24 features due to their SHAP value range being less than 0.2 or their unstable impact as indicated by chaotic color distribution (Figure 3A). The second round further refined the feature set by removing four additional low-contribution features (HGB, WBC, BASO, MPV) from the remaining 14 (Figure 3B).

The final set of 10 retained features (age, RBC, MCV, PDW, LYMPH%, EO%, BASO%, HCT, MONO, MONO%) were both statistically significant and clinically relevant (Figure 3C). These features reflect known risk factors, such as age indicating increased thrombotic susceptibility, RBC linked to blood viscosity, LYMPH% associated with inflammatory responses, and erythrocyte parameters like MCV and HCT related to oxygen-carrying capacity—all recognized indicators of thrombotic risk.

**Figure 4:**
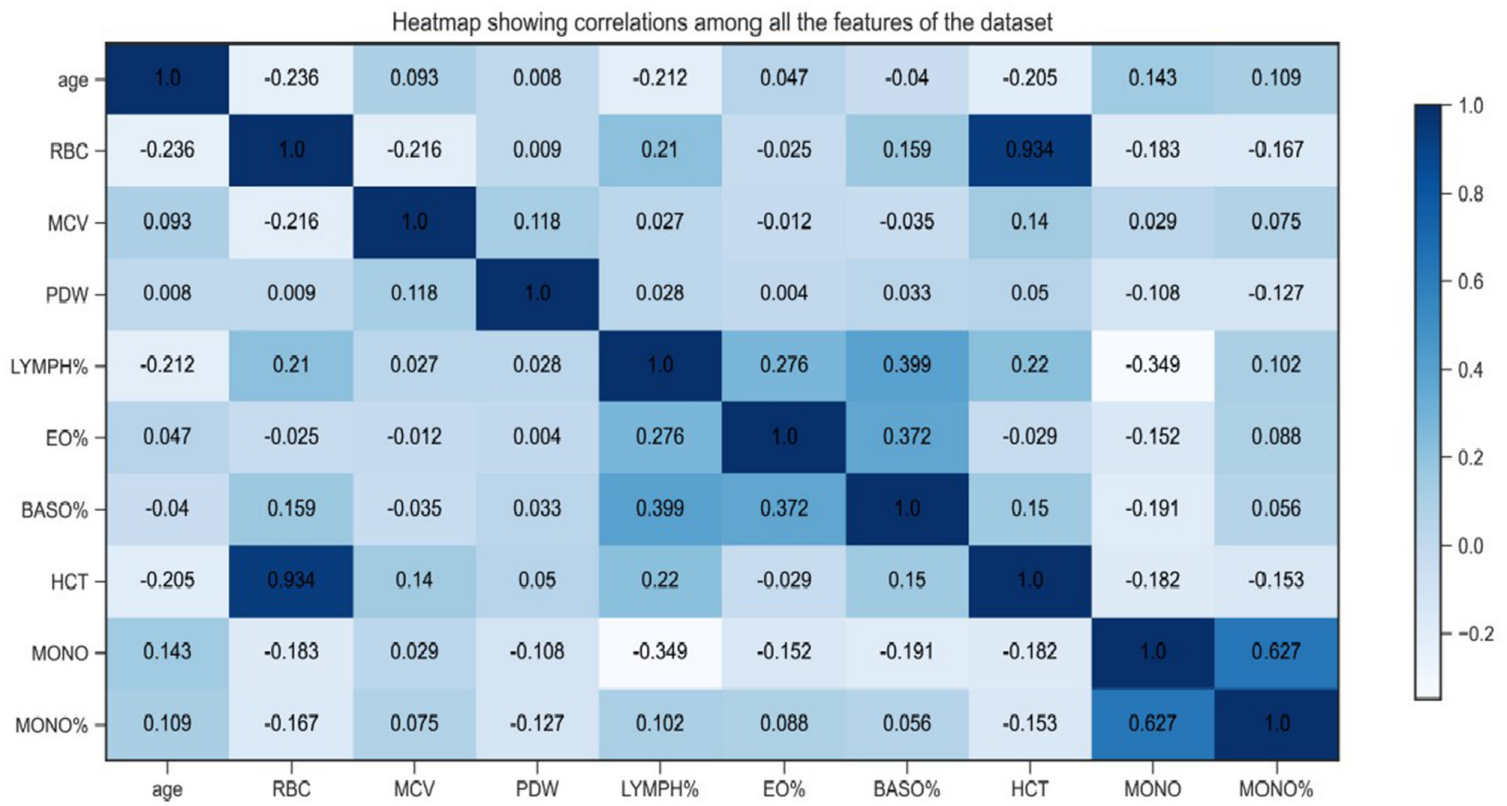
Correlation analysis between features.

Compared to traditional methods, our SHAP-based approach offers advantages: it avoids the pitfalls of filter methods that might miss synergistically important features, wrapper methods that can overfit, and regularization methods like LASSO that may not capture complex relationships. SHAP analysis directly links features to model outputs, enhancing clinical credibility through visual interpretability. The correlation heatmap (Figure 4) confirms the robustness of our selected features, showing no severe multicollinearity (all absolute correlation coefficients < 0.7), thus preventing biases in model predictions.

### 4.3 Results of ML analysis

We preprocessed the DVT dataset and used SHAP analysis to screen out key features affecting DVT risk. As shown in Tables 2, 3, the initial dataset contains various attributes related to patient information and routine blood indicators, laying a foundation for subsequent analyses. Subsequently, we comprehensively compared the performance metrics of multiple classification algorithms (KNN, LR, DT, RF, SVM, and ANN) to identify the optimal prediction model. The model performance was further optimized by adjusting the threshold, and the final model was evaluated on an independent test set.

**Figure 5:**
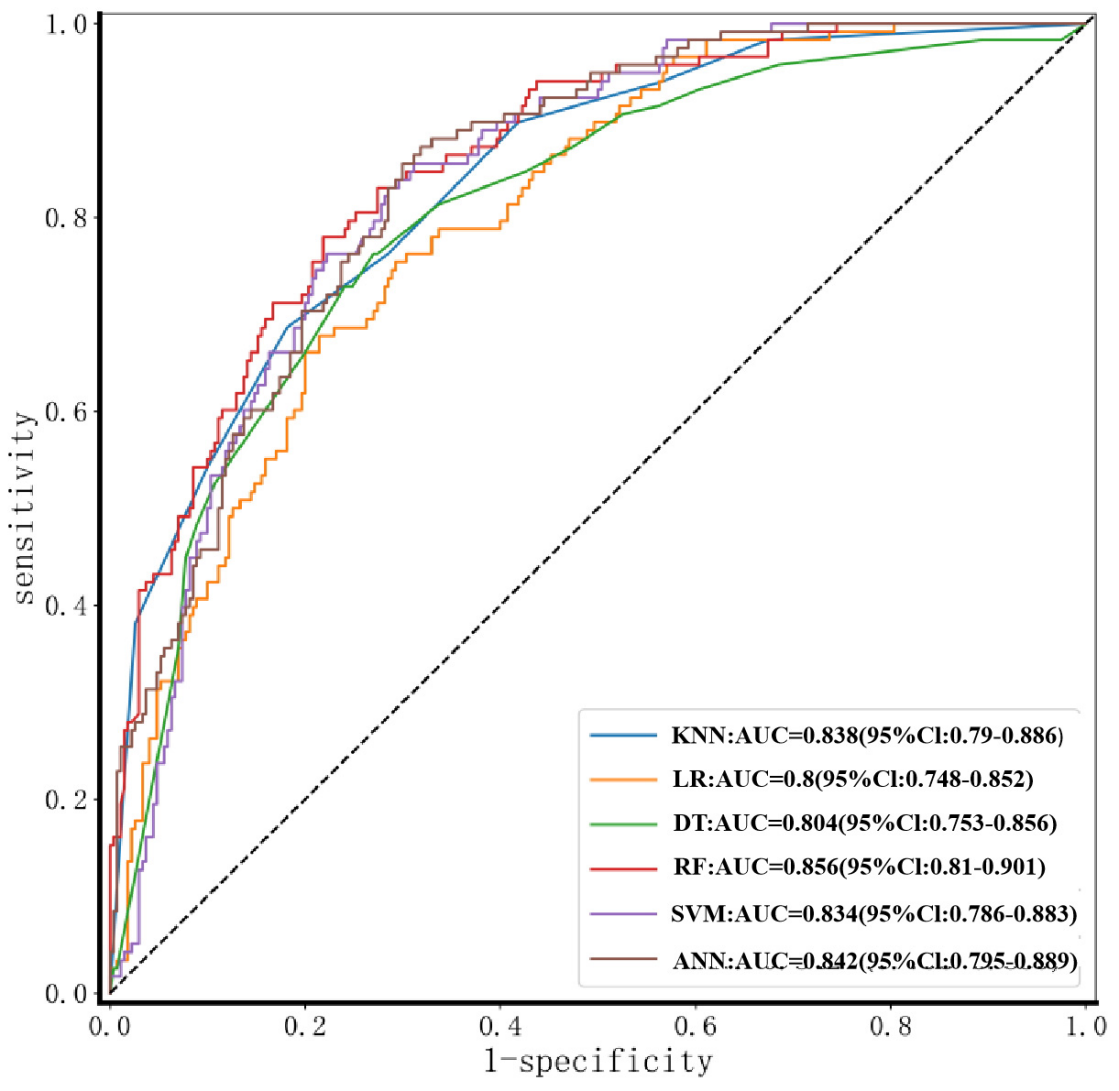
ROC curves of six machine-learning models for DVT prediction. The diagonal gray dashed line indicates the reference of a random classifier (AUC = 0.5). AUC with 95% confidence intervals are listed in the legend. RF achieves the highest discriminative performance (AUC = 0.856).

**Figure 6:**
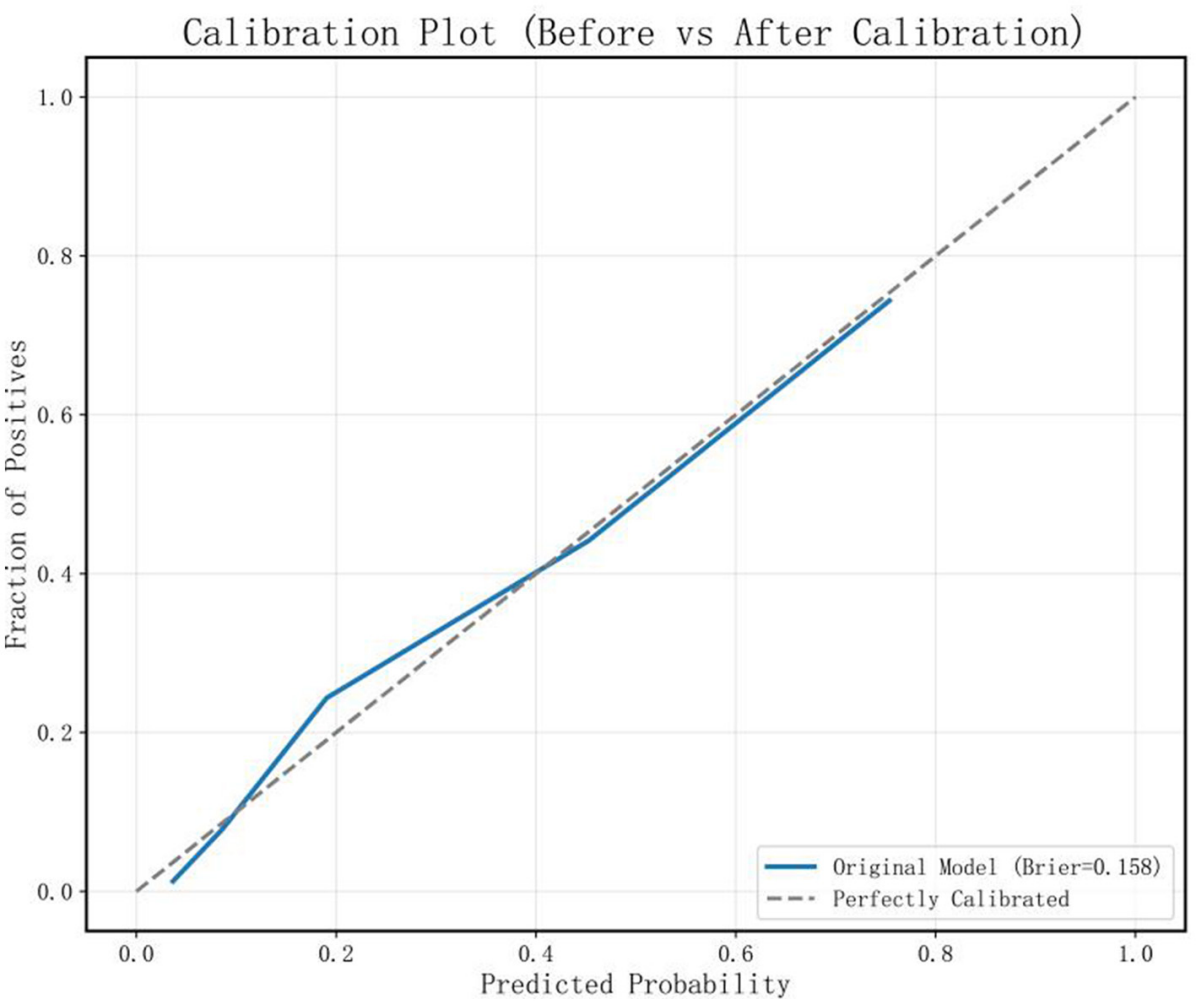
Calibration curve of the random forest model on the validation set with a Brier score of 0.158.

**Figure 7:**
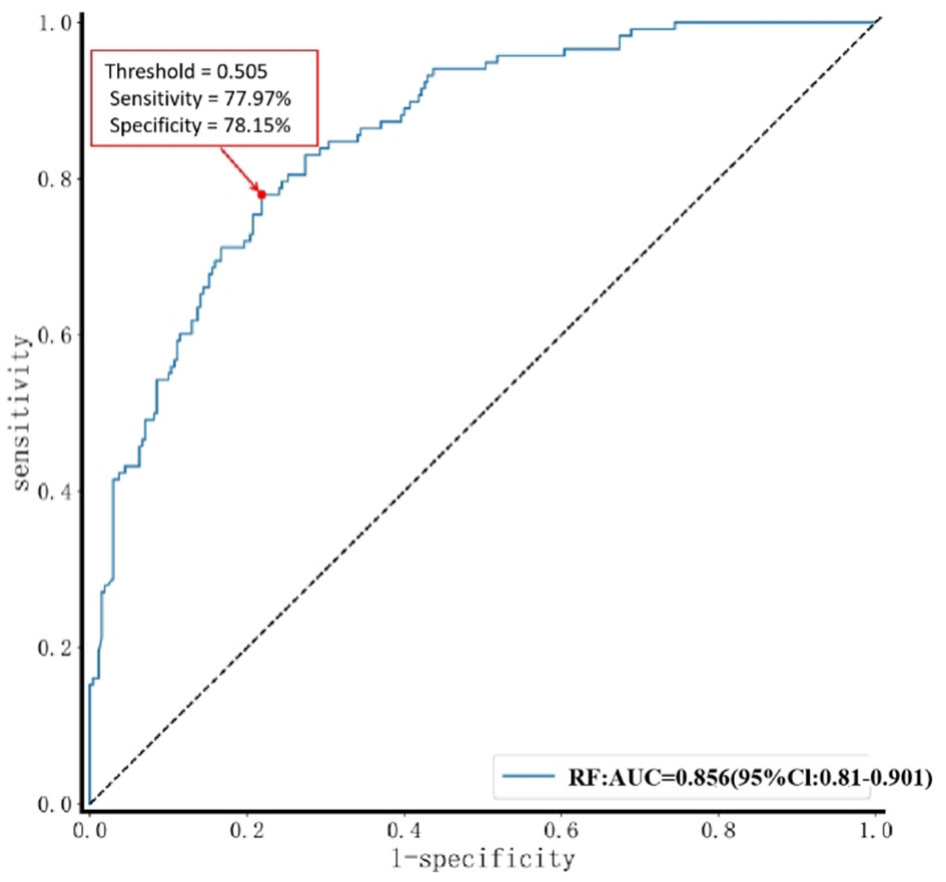
Determination of optimal threshold for RF model. A red arrow indicates the optimal threshold point on the ROC curve. The legend shows that the area under the curve (AUC) of the RF model is 0.856, with a 95% confidence interval of 0.810–0.901. At the optimal threshold marked in the figure, the model achieves a sensitivity of 77.97% and a specificity of 78.15%.

Results from the validation set showed that the RF model performed best across multiple metrics, including accuracy (78.09%), specificity (78.15%), sensitivity (77.97%), AUC (0.856, 95% CI: 0.81–0.901), and F1-score (0.684) (Figure 5, Table 4). The balanced accuracy of this model reached 78.06%. Although all performance metrics were calculated based on the confusion matrix, the specific values of the confusion matrix are not listed in this paper for brevity. Figure 5 provides ROC curves with confidence intervals, reflecting the stability of the model performance. Based on these results, we selected the RF model as the optimal model for assessing lower extremity venous thrombosis risk, with a Brier score of 0.158 (Figure 6). Through ROC curve analysis of the RF model (Figure 7), the optimal classification threshold was determined to be 0.505.

**Table 4:** Performance comparison of different ML algorithms for lower limb DVT prediction.

**Algorithm**	**AUC**	**TN**	**FN**	**TP**	**FP**	**Accuracy**	**Sensitivity**	**Specificity**	**F1-score**	**Balanced accuracy**
KNN	0.838 (0.79–0.886)	193	28	90	77	72.94% (0.685–0.773)	76.27% (0.686–0.84)	71.48% (0.661–0.769)	63.16% (0.584–0.68)	73.88% (0.674–0.804)
LR	0.800 (0.748–0.852)	195	34	84	75	71.91% (0.674–0.764)	71.19% (0.630–0.794)	72.22% (0.669–0.776)	60.65% (0.558–0.655)	71.71% (0.649–0.785)
DT	0.804 (0.753–0.856)	197	28	90	73	73.97% (0.696–0.783)	76.27% (0.686–0.839)	72.96% (0.677–0.783)	64.06% (0.593–0.688)	74.62% (0.681–0.811)
RF	0.856 (0.81–0.901)	211	26	92	59	78.09% (0.74–0.822)	77.97% (0.705–0.854)	78.15% (0.732–0.831)	68.4% (0.638–0.73)	78.06% (0.719–0.843)
SVM	0.834 (0.786–0.883)	202	28	90	68	75.26% (0.71–0.796)	76.27% (0.686–0.839)	74.81% (0.696–0.8)	65.22% (0.605–0.7)	75.54% (0.691–0.82)
ANN	0.842 (0.795–0.889)	207	32	86	63	75.52% (0.712–0.798)	72.88% (0.649–0.809)	76.67% (0.717–0.817)	64.42% (0.596–0.692)	74.78% (0.683–0.813)

**Figure 8:**
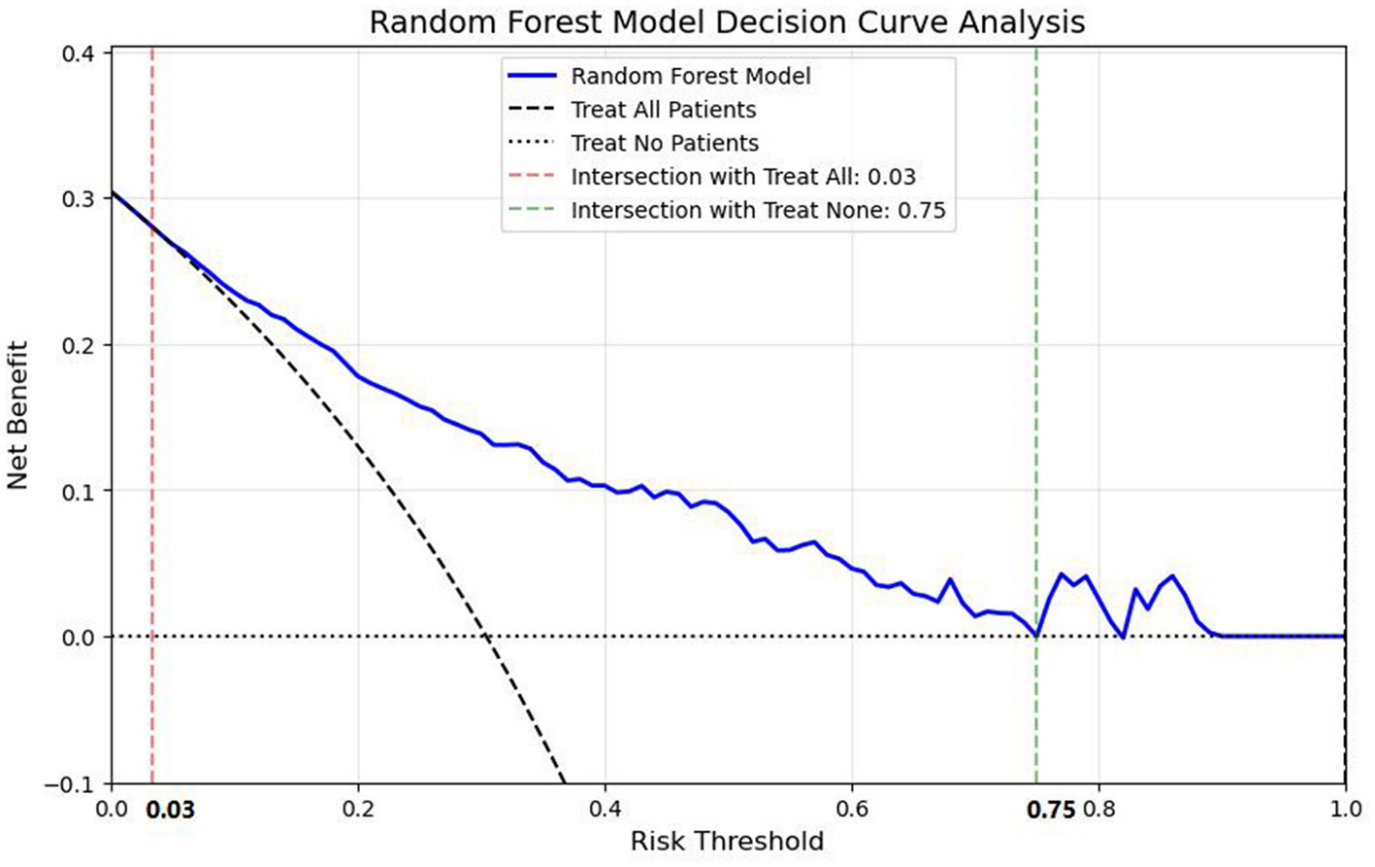
Illustrates the decision curve of the random forest model on the validation set. Within the threshold range of 0.03–0.75, the net benefit achieved by using the model to guide decision-making is consistently superior to that of the two naive strategies, namely predicting all individuals as negative (with a net benefit of 0) and predicting all individuals as positive (with the net benefit corresponding to the “treat all” curve).

Validation on the independent test set confirmed the robust performance of the model, with an AUC of 0.844 (95% CI: 0.778–0.902), TN of 51, FN of 19, TP of 50, FP of 18, sensitivity of 72.46%, (95% CI: 0.62–0.83) specificity of 73.91% (95% CI: 0.635–0.843), accuracy of 73.19% (95% CI: 0.658–0.806), and F1-score of 0.73 (95% CI: 0.656–0.804) (Figure 8). Considering that commonly used clinical thrombosis risk assessment tools (such as the Wells score and Caprini score) may be affected by subjective factors or have limited adaptability to specific populations in practice, the RF model constructed in this study—based on objective patient information and blood indicators—provides a new approach for lower extremity venous thrombosis risk assessment. Future studies can further conduct head-to-head comparisons with existing clinical scoring systems to more comprehensively verify its clinical application value. During model development, we adopted a cross-validation strategy (see Methods section for details) to ensure the model's generalization ability and avoid overfitting.

## 5 Discussion

VTE includes DVT and pulmonary thromboembolism (PTE), which are manifestations of VTE at various locations or stages. DVT forms in the deep veins and its onset is insidious, making it easy to miss or misdiagnose (Connell et al., 2021; Lux et al., 2017; Ruskin, 2018). Routine blood tests and lipid biochemistry indicators can aid physicians in diagnosing DVT, making the relationship between DVT and blood cell components an emerging research focus. Studies have demonstrated that NETs are closely associated with VTE formation (Sun et al., 2020), high hemoglobin (HGB) levels in the general middle-aged population correlate with an increased long-term risk of VTE (Folsom et al., 2020), and MPV is an independent risk factor for mortality post-VTE onset (Díaz et al., 2019).

In the SHAP analysis, we identified 10 key features that contribute significantly to DVT prediction; these indices, characterized by clear physiological significance and clinical interpretability, not only hold the greatest potential for guiding physicians' decision-making but also derive their predictive power from their ability to mirror distinct pathophysiological steps during DVT development. Specifically, RBC count and HCT are directly linked to blood viscosity—higher levels reduce venous flow velocity and create a hemodynamic milieu favorable for thrombus initiation, a conclusion consistent with the findings of (Geerts et al. 2004), who designated HCT >50% as a high-risk criterion for DVT (Geerts et al., 2004). This threshold (HCT >50%) enables physicians to quickly classify patients as high-risk and decide whether to initiate prophylactic anticoagulation (e.g., low-molecular-weight heparin) without relying on more expensive tests like ultrasound. MCV, a measure of erythrocyte size, frequently deviates (< 80 fL or >100 fL) in individuals at increased risk of deep vein thrombosis. This abnormality may indirectly mirror chronic inflammatory states or nutritional deficiencies (e.g., folate or vitamin B12 insufficiency), both of which impair endothelial integrity. A recent large-scale retrospective study in older patients with hip fracture (*n* = 1,840) further demonstrated a linear, positive relationship between admission MCV and preoperative DVT: each 1 fL rise in MCV increased the odds of DVT by 3 % (OR = 1.03; 95 % CI 1.01–1.05; *P* = 0.0013), and patients with abnormal MCV values had approximately 1.5-fold higher DVT risk than those with normal MCV (Xu et al., 2024). For clinicians, this means MCV abnormalities (especially > 100 fL) can prompt closer postoperative monitoring. PDW, a marker of platelet size heterogeneity, rises with enhanced platelet activation; large platelets release more thromboxane A_2_ and pro-coagulant mediators, accelerating the coagulation cascade. Two recent cohort studies showed that each 1 % increment in PDW increased DVT odds by 5 % and independently predicted residual thrombosis and late recurrence (Sevuk et al., 2015; Xu et al., 2024). In clinical practice, a PDW value exceeding the upper reference limit (e.g., >17 fL) often leads physicians to opt for more aggressive anticoagulation regimens or shorten follow-up imaging intervals to monitor for residual thrombus, particularly in patients with a history of DVT recurrence.

A LYMPH% below 20 % indicates a relative lymphocyte deficit and an immunosuppressed phenotype; the resulting imbalance in immune regulation is thought—though not yet proven—to attenuate endothelial repair after vascular injury, thereby potentially increasing the risk of DVT (Nicklas et al., 2020; Zheng et al., 2025). When LYMPH% < 20% is detected in hospitalized patients (e.g., post-surgical or bedridden individuals), physicians may adjust care plans to reduce DVT risk, such as encouraging early ambulation or adding mechanical prophylaxis (e.g., intermittent pneumatic compression) alongside pharmacological measures. EO% (>5%) or BASO% (>1%) may promote vascular endothelial injury and local inflammatory responses through the release of cationic proteins from eosinophils and histamine from basophils, thereby contributing to thrombus formation. Recent clinical studies have also indicated a significantly higher positive diagnostic rate of DVT in populations with increased proportions of eosinophils or basophils (odds ratio [OR] = 1.92). However, due to their weaker predictive power compared to other indices, EO% and BASO% are typically used as auxiliary references—physicians may consider these values when other high-risk features (e.g., elevated PDW) are present, but rarely base standalone anticoagulation or screening decisions on them. The roles of MONO and MONO% are particularly crucial: monocytes can differentiate into macrophages to participate in inflammatory responses and also express tissue factor (TF) to activate the extrinsic coagulation pathway. The 2020 VTE Clinical Practice Guidelines by the American Society of Hematology (ASH) emphasize that when MONO exceeds 0.8 × 10^9^/L, the expression level of tissue factor doubles. Additionally, a study by (Wang et al. 2023) has also confirmed that monocytes can participate in the thrombus stabilization process through interacting with neutrophil extracellular traps (NETs) (Han et al., 2023). In line with this evidence, physicians often use MONO > 0.8 × 10^9^/L as a trigger for intensified DVT screening—for example, ordering a lower-limb venous ultrasound for patients with prolonged bed rest (≥ 72 h) and elevated MONO, even in the absence of obvious clinical symptoms like leg swelling.

ML algorithms aim to identify linear or nonlinear functions for classification or prediction tasks (Sorich et al., 2003). We selected six algorithms—KNN, LR, DT, RF, SVM, and ANN—each with specific advantages. Our dataset, comprising 10 features and 2076 data entries, has a low feature dimension. Adopting current deep learning methods would risk overfitting due to the complex structure and numerous parameters of deep learning models in scenarios with small sample sizes and low-dimensional data. Overfitting results in poor generalization to new data, impeding the desired high-performance prediction outcomes. Thus, we chose these six machine learning models to effectively capture the relationship between data features and DVT diagnosis under limited data conditions while maintaining good generalization performance and prediction accuracy (1) KNN, selected for its simplicity and intuitiveness, KNN makes predictions based on sample similarity without complex training processes, making it suitable for data with clear local structures. For instance, in datasets where samples cluster in specific patterns, KNN can swiftly classify new samples according to the proximity of surrounding samples (Kamran et al., 2022). (2) LR, a generalized linear regression analysis model, LR is popular in medical research due to its excellent interpretability in binary classification problems. In predicting DVT risk, it clearly shows how each feature contributes to the probability of DVT occurrence, providing valuable insights for clinical decision-making (Lunt, 2015). (3) DT, predicting attributes based on tree-shaped decision rules, DT handles both numerical and categorical data. Its tree-like structure makes the decision-making process transparent and easy to understand, facilitating the analysis of relationships between various blood indicators and DVT risk by revealing hierarchical and conditional relationships within the data (Khan et al., 2010). (4) ANN, categorized as deep supervised learning, ANN has a powerful ability to learn complex patterns, automatically discovering hidden relationships among various factors that are difficult to identify using traditional algorithms (Wu et al., 2022). (5) SVM, a data classification method based on hyperplane multidimensional data sorting, SVM effectively handles high-dimensional data. In the field of medical data analysis, where numerous features are common, SVM can find the optimal hyperplane to separate different classes and use kernel functions to handle non-linearly separable data, making it suitable for dealing with the complex relationships in medical datasets (Janardhanan et al., 2015). (6) RF, a modeling method based on decision trees without assuming a data distribution, RF overcomes the limitations of a single decision tree. By aggregating multiple decision trees, it reduces variance and overfitting issues, offering better generalization ability and effectively capturing complex patterns in medical data, which often contain a large number of variables and non-linear relationships (Breiman, 2001).

In our study, all six algorithm models achieved AUCs above 0.8, accuracy rates above 70%, and sensitivities and specificities above 70%. The RF model performed the best in assessing the risk of lower-extremity venous thrombosis, with an AUC exceeding 0.85, demonstrating relatively good performance and clinical application potential in the field of disease screening.

Existing DVT prediction methods have notable limitations. Traditional clinical scoring systems, such as the Wells score and Caprini score, rely heavily on subjective symptom judgments and specialized, often expensive, examinations like ultrasound and D-dimer tests. DVT prediction models have now demonstrated good performance in clinical practice. For instance, a nomogram model developed for patients with hip fractures—incorporating indicators such as age and surgical method—achieves AUC of 0.815, which is significantly superior to the Caprini score's AUC of 0.659 (Bo et al., 2024). Additionally, based on 11 indicators that showed differences between the DVT group and non-DVT group among patients undergoing laparoscopic abdominal surgery, the study selected 5 superior machine learning algorithms and constructed the LASDVT model through stacking integration. The core predictive factors of this model included tumor history and age; after external validation in 114 patients, the model demonstrated excellent performance (AUC = 0.9293), and its predictive effect was significantly superior to that of the Caprini score (*p* = 0.0047) (Yang et al., 2025). Most current ML models further exacerbate the cost issue by incorporating costly indicators like imaging data or molecular markers. In contrast, our study makes significant improvements. We innovatively constructed a DVT prediction model using only routine clinical test items, specifically blood routine tests—this circumvents high costs, enabling early risk assessment even in resource-limited settings such as rural clinics where advanced diagnostics are scarce.

### 5.1 Strengths and limitations

Our study's key strengths lie in its accessibility and interpretability: using only routine blood tests eliminates cost barriers to early screening, while SHAP analysis clarifies how features like platelet parameters contribute to predictions, aligning with clinical knowledge and enhancing physician trust. These innovations offer a practical tool for widespread use, from tertiary hospitals to community clinics.

The study, however, has several limitations. First, there is a lack of direct performance comparison with clinical standard scoring systems. The currently widely used Wells score (for initial DVT screening) and Caprini score (for VTE risk stratification in inpatients) serve as “benchmark tools” for DVT risk assessment. However, this study did not compare the constructed model with these two scores on the same dataset through direct quantitative analysis (e.g., comparison of AUC, sensitivity, and specificity). This omission prevents a clear determination of the model's comparative advantage over traditional clinical tools and hinders clinicians from intuitively evaluating its substitutive or supplementary value. For example, the specific extent to which the model improves the reduction of subjective judgment bias (such as the subjective assessment of “leg swelling” in the Wells score) or decreases reliance on D-dimer testing (such as the requirement to integrate D-dimer results for partial risk stratification in the Caprini score) remains unclarified. Second, the single-center, retrospective design and selection of a specific population severely limit the generalizability of the study. The study data were collected retrospectively from a single institution, Baoding Second Central Hospital, and the cohort was strictly restricted to “surgical inpatients aged 50–80 years”—these factors collectively compromise the external validity of the model: (1) Blood routine parameters vary by region. Indices differ between Baoding's population and those in southern humid or western plateau areas (e.g., higher HCT in plateau populations due to hypoxia). Directly applying DVT risk thresholds calibrated for Baoding may lead to underdiagnosis or overdiagnosis, and regional differences in underlying diseases (more hypertension in northern China, more diabetes in southern China) and lifestyles further amplify such biases. (2) Testing standards are inconsistent. This study used the Sysmex platform, while some primary hospitals use the Beckman Coulter platform. Differences in platforms, reagents, and reference ranges may cause inconsistent results for the same patient, making the model's “risk judgments” (trained on Sysmex data) less applicable in institutions with other platforms. (3) The selection of “surgical patients aged 50–80 years” is reasonable, as this group accounts for 61.5% of DVT cases in the hospital and belongs to a high-risk population; however, subgroups such as pregnant women and cancer patients were excluded from the study. Abnormal blood routine results in these excluded subgroups are mostly driven by their own physiological or pathological states, which may interfere with the analysis of the association between blood routine parameters and DVT. Additionally, these subgroups constitute < 20% of all DVT cases, and their inclusion in the study could introduce biases. This “targeted selection” approach results in the model being unable to cover these subgroups, thereby limiting the model's use in obstetrics, oncology, and among younger patients.

To address the limitations of the current study and enhance the model's reliability, future work should focus on four key directions: firstly, synchronously collecting Wells score and Caprini score data in a multi-center cohort (*n* = 5,000) to conduct direct performance comparisons (e.g., AUC, sensitivity, specificity) between the model and these traditional scoring tools, thereby clarifying the model's clinical advantages and positioning; secondly, collaborating with 8–10 tertiary hospitals and 15–20 primary healthcare institutions across eastern, southern, western, and northern China (e.g., Shanghai, Guangzhou, Xining), and incorporating data from diverse populations such as plateau residents, Tibetans, and Uyghurs to validate the model's stability across different geographical regions and ethnic groups while calibrating regional differences in blood routine parameters; furthermore, establishing calibration equations across mainstream blood testing platforms (e.g., Sysmex, Beckman Coulter) using standard samples to validate the model's consistency across multiple devices and improve its universality; finally, conducting targeted validation for key subgroups including pregnant women, cancer patients, and young patients with inherited thrombophilia to optimize the model's prediction thresholds for these populations, and adjusting the model's performance based on hospital tiers—prioritizing sensitivity enhancement in primary healthcare institutions to reduce missed diagnoses, while balancing sensitivity and specificity in tertiary hospitals to avoid over-medicalization.

Addressing these limitations through the above broader validation plans could significantly enhance the robustness and applicability of our DVT prediction model, ultimately contributing to improved patient outcomes and reduced healthcare costs.

## 6 Conclusions

DVT is a major global contributor to preventable death and morbidity. Leveraging data mining and machine learning, we identified 9 routine blood features (including RBC, PDW, and MONO%) as key predictors of DVT risk. Among tested algorithms, the RF model outperformed others with an accuracy of 78.09% and AUC > 0.85.

This study's novelty lies in its dual focus on accessibility and interpretability: by using only routine blood data, the model eliminates cost barriers to early screening, while SHAP analysis links predictions to pathological mechanisms, enhancing clinical trust. Its potential impact includes reducing missed diagnoses in primary care and optimizing resource allocation (e.g., prioritizing ultrasound for high-risk patients).

Future work will refine the model through multi-center validation and integration of temporal variables, aiming to establish it as a standard tool for DVT risk assessment across diverse clinical settings.

## Data Availability

The datasets presented in this article are not readily available because 1. Data Source Limitation: The dataset was collected from patients hospitalized at the Baoding Second Central Hospital. It is sourced from a single center, which may lead to selection bias. As a result, the data may not be representative of the entire population with deep vein thrombosis (DVT) or the general population, limiting the generalizability of the findings. 2. Sample Inclusion and Exclusion Criteria: The dataset includes patients aged between 50 and 80 years old, hospitalized in general or thoracic surgery departments, and without other serious cardiovascular diseases or bleeding tendencies. Exclusion criteria include the lack of complete routine blood data and other factors affecting hemorheology. These specific criteria narrow down the scope of the dataset and may not cover all possible patient scenarios related to DVT. 3. Diagnosis Reliance: Since the diagnosis of DVT mainly relies on imaging examinations, there may be missed diagnosis cases that have not been included in the dataset. This affects the integrity and accuracy of the data, as some patients with actual DVT might be absent from the dataset, leading to potential inaccuracies in the analysis and model building. 4. Influence of Confounding Factors: Although SHAP analysis was used for feature selection, some variables in the dataset may be influenced by factors such as inflammatory status, malignant tumors, or drug use. The model has not explicitly adjusted for these confounding effects nor modeled the interactions between features, which restricts the accuracy of causal inference based on the dataset. 5. Absence of External Validation: The dataset has only been used for internal validation within a single center. It has not been tested on patients from different regions, ethnic groups, levels of healthcare resources, or special subgroups. This lack of external validation limits the model's ability to be applied in various clinical scenarios and reduces its overall reliability. Requests to access the datasets should be directed to Biao Song, songbiao_511@163.com.
